# Prevalence of Infectious Diseases in Children at Preschool Education Institutions and Stakeholder Opinions

**DOI:** 10.3390/children11040447

**Published:** 2024-04-08

**Authors:** Gonca Kurt, Hasan Uğur Serdaroğlu

**Affiliations:** 1 Department of Health Care Services, Pazar Vocational School of Higher Education, Tokat Gaziosmanpaşa University, 60800 Tokat, Türkiye; 2 Department of Childhood Development, Pazar Vocational School of Higher Education, Tokat Gaziosmanpaşa University, 60800 Tokat, Türkiye; huserdaroglu@gmail.com

**Keywords:** preschool, infectious disease, teacher, parent, presenteeism, early childhood, respiratory diseases, diarrheal diseases, skin rashes, antibiotics

## Abstract

Preschool education institutions, where children have close contact and social interactions, can serve as potential environments for the transmission of infectious diseases. This issue poses a significant health concern, impacting both individual and public well-being. Thus, the present research set out to determine the prevalence of infectious diseases in preschool children and the views of parents and teachers on the prevention of infectious diseases. The study employed a mixed-method approach and involved 397 parents and 46 preschool teachers. The study was survey-based via in-person interviews. The results revealed that after they started school (almost in 5 months), children with a mean age of 4.7 ± 0.5 years experienced illness approximately 3.5 ± 2.0 times, of whom 91.5% used antibiotics. The prevalence of infectious diseases was found to be associated with the child’s being born at term, maternal education level, and the public/private status of preschool institutions. The presence of a sick child in the classroom elicits negative emotions from both teachers and parents. They recommend that studies on education, safety, hygiene, school health, health screenings, regulation of the learning environment, legal regulations, and school exclusion policies be carried out to prevent infectious diseases. When children with infectious diseases come to school, nearly half of the teachers admit them to the classroom due to various reasons and pressures. Parents request teachers to monitor medication, control sweating, and use a nebulizer for their sick children. Some of the teachers meet these requests, but they claim that the educational process is negatively affected. At preschool education institutions, the risk factors of infectious diseases have a complex structure and can be influenced by variables related to teachers, children, parents and the institution itself. Infectious diseases not only pose a threat to health but also impact teachers’ and parents’ emotions, teacher–child relationships, and the overall atmosphere within schools and classrooms.

## 1. Introduction

The Industrial Revolution in Europe in the 18th century led to a notable increase in women’s labor force participation, further amplified in the aftermath of World War II, resulting in a significant rise in female employment. Their participation in the labor force has affected family structures, and thus, the trend toward a nuclear family structure has advanced, shifting from extended families that assume the caretaking responsibility of children in the absence of parents. Due to these developments, preschool institutions have gained prominence in fulfilling the basic needs and providing care for working parents’ children. The primary purpose of preschool education is to support children’s cognitive, language, motor, and social-emotional development and to promote their self-care skills. They are provided with health-related knowledge and awareness, and positive attitudes and behaviors are fostered in line with this objective. The public’s recognition of the significance of early childhood education has further increased the rate of children receiving education from preschool institutions. According to the Organization for Economic Co-Operation and Development (OECD) and the Ministry of National Education of the Republic of Türkiye data for 2023, the preschool enrollment rates in Türkiye have reached 21% for 3 year-olds, 42% for 4 year-olds, and 99.86% for 5 year-olds [1,2]. Enrollment rates in early childhood education are also increasing in regions such as America, Europe, Scandinavia, and Oceania [3]. Although this increase is a highly valuable advancement, it has also brought forth certain risks.

Preschool institutions, where children have close contact and social interactions, can cause the transmission of infectious diseases, whereas they contribute to their development and immunization. Nesti and Goldbaum stated that the risk of infection among children at preschool education centers has increased 2–3 times, which is a significant issue in terms of both personal health and community health [4]. Several other studies indicate the prevalence of infectious diseases and the increased risk of infection at preschool education institutions [5,6,7,8,9,10,11,12,13,14,15,16]. They are identified as a risk factor in the spread of infectious diseases; however, the solution to this risk does not involve keeping children away from them. The way to reduce the prevalence of infectious diseases is to pinpoint the causes of transmission and its risk factors. By thoroughly investigating the causes of infectious diseases and considering the viewpoints of all stakeholders, suggestions for solutions can be proposed to create healthier and safer preschool education environments.

Limited research has been conducted on infectious diseases which are becoming increasingly prevalent at preschool institutions, posing a growing risk to public health and creating economic burdens. They primarily focused on the factors influencing parents’ decisions to send their children to school despite being infected. These studies have identified factors such as employment pressures, financial constraints, and school policies as influential in parental decision-making [8,9,13,16]. The reasons for the spread of infectious diseases at preschool institutions are so involved that they cannot be attributed solely to parental factors. A number of factors with distinct variables affect this situation, among which the most crucial one regards teachers, such as their attitudes towards parents and sick children, educational level, chronic disease status, age of the children they instruct, and whether they work at a private or public institution. Other factors are related to the children (chronic disease status, duration of formula milk intake, type of birth, number of siblings, vaccination status, exposure to cigarette smoking, and regular use OF medications), parents (educational status, occupation, income level, smoking status, and alcohol consumption during pregnancy), and institutions (student exclusion policy, the maximum duration of absence, regulations, school nutrition environments, school meals, ventilation, school district size, temperature, number of staff employed, sterilizing practices). A search of the literature revealed that studies on this topic are quite limited, and no previous research has included teachers who play a vital role in shaping the decisions of children with infectious diseases to attend preschool institutions and implementing student exclusion policies. Indeed, their attitudes toward these children and their parents may affect parental behavior and decisions. A comprehensive study addressing all the factors regarding teachers, parents, children, and schools can specify the causes of infectious diseases at preschool institutions and contribute to potential solutions. The lack of research and solutions to this issue will not prevent the rapid increase in and spread of infections at preschool institutions.

Infections are a major cause of morbidity and mortality among preschool children [17,18,19]. With regard to the reports published by the United Nations International Children’s Emergency Fund and the ones published in Türkiye in 2023, infections are among the leading causes of death of children younger than 5 years [20,21]. Specifically, respiratory tract infections, diarrheal diseases, and contagious diseases characterized by rashes, such as chickenpox, mumps, measles, and rubella, can spread rapidly within the school environment. In preschool settings, such diseases pose a crucial public health concern and entail a financial burden. Investigating the causes of disease transmissions and accordingly taking precautions are of vital importance for children’s health, quality of life, and academic achievement since both children’s health and education are negatively affected. Preschool institutions prioritizing and maintaining higher health and safety standards can positively impact school staff, families, society, the economy, and especially children. Moreover, preventing the spread of infectious diseases at preschool institutions can mitigate the risk of parents experiencing job loss, economic loss due to medical expenses, and unnecessary antibiotic use. In this respect, extensive and comprehensive research is needed to encompass various aspects, including the learning environment, school policies, and parents, in order to prevent infectious diseases at preschool institutions effectively [7,9,15,16,22,23]. Furthermore, a new concept, school-based presenteeism, defined as the sick child attending school for a period, was introduced into the literature in 2023, and the imperative to conduct further studies on the factors affecting it was underlined [24].

Hence, this research set out to investigate the prevalence of infectious diseases, including respiratory tract infections, diarrheal diseases, and rashes, along with the underlying causes of their transmission within preschool education institutions. By conducting a comprehensive study encompassing all variables related to teachers, children, parents, and institutions, the current study aimed to contribute to developing intervention programs to prevent the increase in and spread of infectious diseases.

## 2. Materials and Methods

### 2.1. Participants and Setting

This cross-sectional research was conducted in the Tokat province of Türkiye between November and December 2023. The study sample involved teachers working at preschool institutions in Tokat and parents with preschool children. According to data from the Ministry of National Education of Türkiye, there were 49 preschool institutions in Tokat city center during the 2023–2024 academic year. The number of teachers and students at these institutions was 175 and 4733, respectively. In the present study, sample size calculation was not performed as it was sought to reach the entire study population on a voluntary basis. The researchers first went to each pre-school education institution in the province and reached the teachers who agreed to participate in the study. Then, parents were reached through the school administration and teachers. Finally, the research was conducted with 46 teachers and 397 parents. All the teachers participating in the study were female, whose mean age was 39.8 ± 4.2 years, and the majority had 11 years or above of teaching experience (Supplementary Materials Table S1). To be eligible to teach at preschool institutions in Türkiye, one must graduate from the Department of Preschool Education of the Faculties of Education. Parents partaking in the study comprised 369 mothers (92.9%) and 28 fathers (7.1%), whose socio-demographic characteristics are demonstrated in Table S2. Data were obtained from parents to identify the prevalence of infectious diseases in children attending preschool institutions, which is the primary purpose of the research. Most children (77.1%) were five years old and were born by cesarean section (62.5%). Of the children, 7.3% had a chronic disease (n = 29); the most common was asthma (72.4%, n = 21). Other information about children is presented in Table S3.

### 2.2. Data Collection

This research adopted a convergent mixed-method design, wherein qualitative and quantitative data are combined with the objective of generating a comprehensive understanding of the research problem [25]. The researchers collected the data through a literature review that was aligned with the study’s aim. Face validity was a key consideration during the creation of survey questions [26]. Three field specialists—an infectious diseases specialist, an academic from the Department of Preschool Education, and a preschool teacher with 12 years of experience—were consulted to ensure that each survey question reflected the research purpose and adequately covered the scope of the study. Based on their feedback, the final version of the data collection tools was created. Moreover, in order to test the functionality of the questionnaire questions, we applied the questionnaire questions to 5 parents and 2 teachers. As a result of the pre-application, no changes were made. Consequently, the research data were gathered via two semi-structured questionnaires: ‘Infectious Diseases in Preschool Education Institutions: Questionnaire for Parents’ and ‘Infectious Diseases in Preschool Education Institutions: Questionnaire for Teachers’. The former consisted of two sections. The first section contained 24 questions regarding children’s demographic information, health, and infectious diseases. The second section consisted of 24 open- and closed-ended questions about parents’ socio-demographic characteristics, attitudes, and behaviors toward sending a preschool child to school in case of infectious disease, as well as their suggestions for solutions to ensure control of children with a contagious disease at schools. The data from the parents were collected through face-to-face interviews or online questionnaires, depending on the parents’ requests. The questionnaire regarding teachers consisted of demographic questions and 27 open- and closed-ended questions addressing the challenges encountered in classroom management with a student with an infectious disease, potential courses of action, and suggestions for solutions. The data for teachers were gathered via face-to-face interviews.

### 2.3. Statistical Analysis

#### 2.3.1. Quantitative Data Analysis

Regarding descriptive statistics, quantitative data were presented as the means and standard deviations, and qualitative data were given as numbers and percentages. The dependent variable of the research was the number of children getting sick after they started school. The data obtained on the frequency of infectious diseases in children are based on the reports of parents. The independent variables constituted children’s health conditions, age, birth order, number of siblings, type and time of birth, duration of breastfeeding, presence of chronic disease and allergies, full vaccination coverage, and type of school. Parent-related variables were also involved in the study, such as smoking status during pregnancy, presence of parents who smoke at home, age, occupation, and socio-economic status. When there were two independent groups, the *t*-test was performed for independent samples to compare quantitative variables. When there were more than two independent groups, a one-way analysis of variance (ANOVA) was utilized. Once any significant difference was found between at least two independent groups as a result of one-way ANOVA, the Tukey test (assumption of homogeneity of variance), a post hoc test, was employed to determine the groups that showed statistically significant differences. The statistical significance was set at *p* < 0.05. The research data were analyzed using IBM SPSS Statistics, Version 21.

#### 2.3.2. Qualitative Data Analysis

Thematic analysis was applied to the answers given to the open-ended questions [27], and a six-stage process was used to analyze the data. First, the researchers created initial notes upon reading the data. Second, emerging themes were systematically coded (emotions, causes of infectious diseases, attitudes toward a sick child, experiences, and suggestions for solutions to prevent infectious diseases), and the data related to these codes were combined. Third, sub-themes and themes regarding the codes were created, followed by an evaluation of the consistency of the identified themes with the data. During this process, the themes’ compatibility with the data content and dataset were considered. Afterward, the themes were defined and named. During the final stage, a report was prepared using quotes reflecting the participants’ opinions. Of the transcripts, 20% were independently coded by two researchers (G.K. and HU.S.). Any discrepancies in coding and thematic interpretation were resolved by consensus. The complete transcripts were then made. The software MAXQDA 24 was used to analyze and report the qualitative data.

In this study, the structures proposed by Lincoln and Guba were emphasized to ensure validity and reliability [28]. That is, the researchers focused solely on the data, avoiding the influence of their own biases. Objectivity and transferability were also achieved by presenting the participants’ statements as direct quotations.

## 3. Results

### 3.1. Data on the Prevalence of Infectious Diseases in Children at Preschool Institutions and Their Parents’ Views

In this study conducted with preschool children’s parents, the mean age of the children was found to be 4.7 ± 0.5 years (Table S3). Of the parents, 47.9% reported that their children get sick 3–5 times a year. As for 23.2% of the parents, their children get sick more frequently than their peers. More than half of them (57.7%) believed that their children become ill more often on school days. From the start of the school year to the time of this study (approximately five months), the frequency of infectious diseases in children was identified as 94.7%, and children were sick with a mean of 3.5 ± 2.0 times (Table 1). In the comparison of the frequency of sickness after the start of school, which serves as the dependent variable in the study, with independent variables, it was found that children who attended a private preschool institution (4.2 ± 2.1; *p* < 0.031) and an independent kindergarten (3.8 ± 2.1; *p* < 0.025), and those whose mothers had high school and a master’s or doctoral degree (3.9 ± 2.1; 4.4 ± 2.4; *p* < 0.010, respectively) exhibited a significantly higher rate of illness compared to the other groups. Term births (3.4 ± 1.9) were less likely to become ill than premature and postmature births. No statistically significant difference was observed between the dependent variable and the other independent variables (*p* > 0.05) (Table S4).

While the incidence of infectious diseases in the last two weeks was 61.7%, respiratory tract infections were the most common. The majority of the sick children (91.5%) used antibiotics prescribed by the doctor (Table 1).

### 3.2. Parents’ Attitudes and Behaviors toward Sending Their Children with an Infectious Disease to School

Of the parents, 55.2% believed that they were knowledgeable about childhood contagious diseases, and 4.8% of them reported that they send their sick children to school since nobody can care for their children during work hours, which was noted as the primary reason. The majority (81.4%) also thought their children get sick at school (Table 2).

### 3.3. Parents’ Views on Infectious Diseases at Preschool Institutions (Qualitative Data)

**Figure 1:**
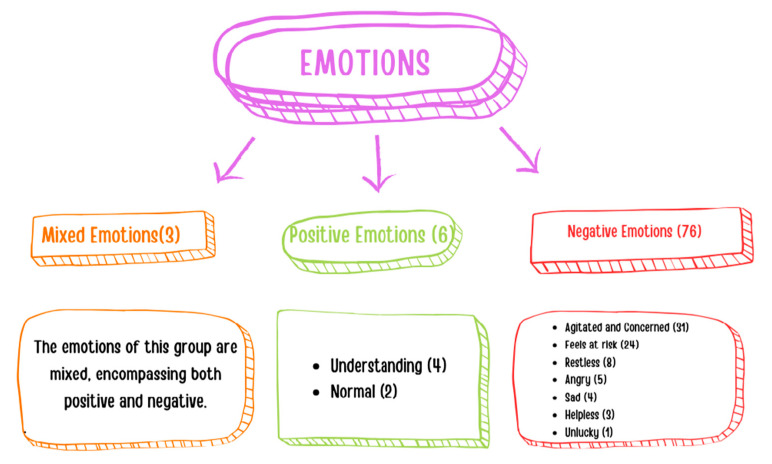
Parents’ emotions when a child with an infectious disease attends the classroom.

Parents were asked to describe their emotions when a child with an infectious disease attended the classroom where their child also receives education. They reported experiencing predominantly negative emotions (n = 76), among which the most prevalent ones were agitated and concerned (n = 31), as well as feeling at risk (n = 24). The number of parents experiencing positive emotions (n = 6) and mixed emotions (n = 3) was low (Figure 1).

*“……In such a situation, I believe my child and I are at risk. I do not want to send my child to school for protection.”* (Parent-226).

### 3.4. Parents’ Views on the Causes of Infectious Diseases at Preschool Institutions (Qualitative Data)

**Figure 2:**
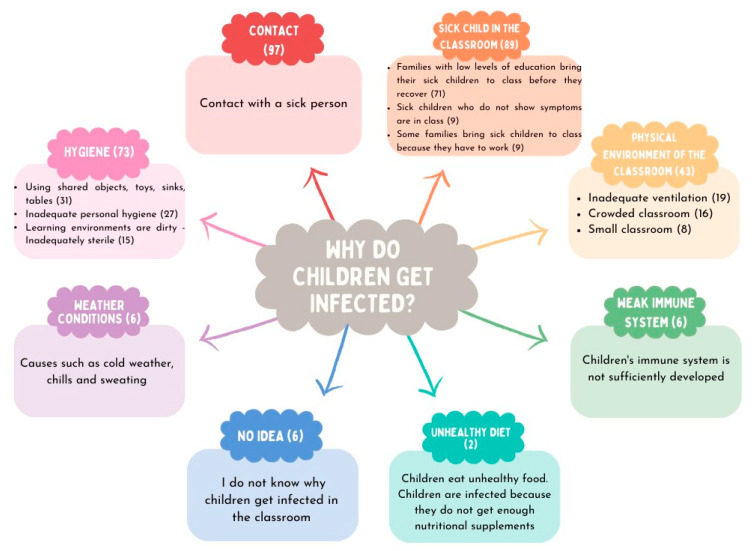
Parents’ views on the causes of infectious diseases at preschool institutions.

Parents were asked why they think their children contract an infectious disease at preschool institutions. Their responses included contact with a sick person (n = 97), presence of a sick child in the classroom (n = 89), hygiene issues (n = 73), physical classroom environment (n = 43), weather conditions (n = 6), weak immune system (n = 6), and unhealthy diet (n = 2). Some of them, on the other hand, declared that they had no idea (n = 6) (Figure 2).

### 3.5. Parents’ Views on the Prevention of Infectious Diseases at Preschool Institutions (Qualitative Data)

**Figure 3:**
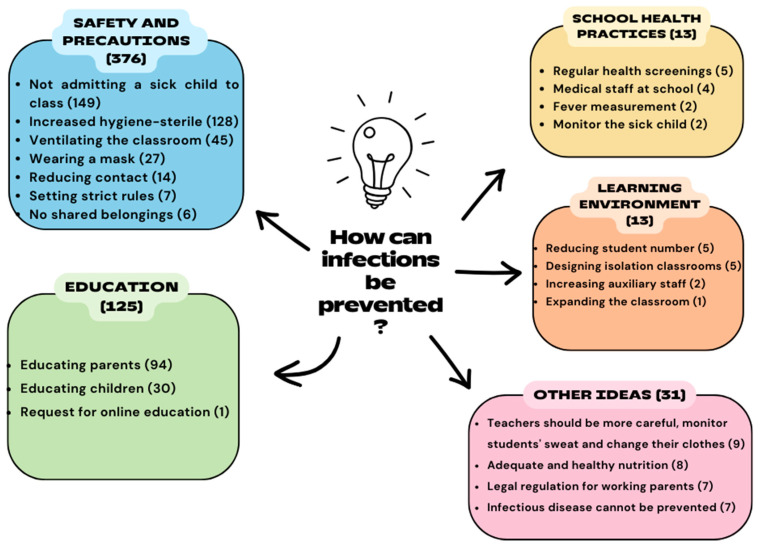
Parents’ views on the prevention of infectious diseases at preschool institutions.

Parents were asked to comment on how infectious diseases at preschool institutions can be prevented. They emphasized improving safety and precautions (n = 376), education (n = 125), school health practices (n = 13), organizing the learning environment (n = 13), and other ideas (n = 31). A few participants in the last category (n = 7) remarked that infectious diseases cannot be prevented and, thus, taking precautions would not be efficient (Figure 3).

*“Infection cannot be prevented. Children must get sick, and thus their immunity must be strengthened”* (Parent-108).

### 3.6. Teachers’ Views on Infectious Diseases at Preschool Institutions and Their Experiences

Of the 46 participating teachers, 40 declared that they knew about childhood infectious diseases. The number of teachers who reported that their students with an infectious disease attended the classroom was 19. The most commonly encountered type of disease was respiratory tract infections (Table 3). All teachers concurred that sick children should not be sent to school. They also mentioned that they cautioned parents about this issue, as it could result in other students becoming infected with the disease.

**Figure 4:**
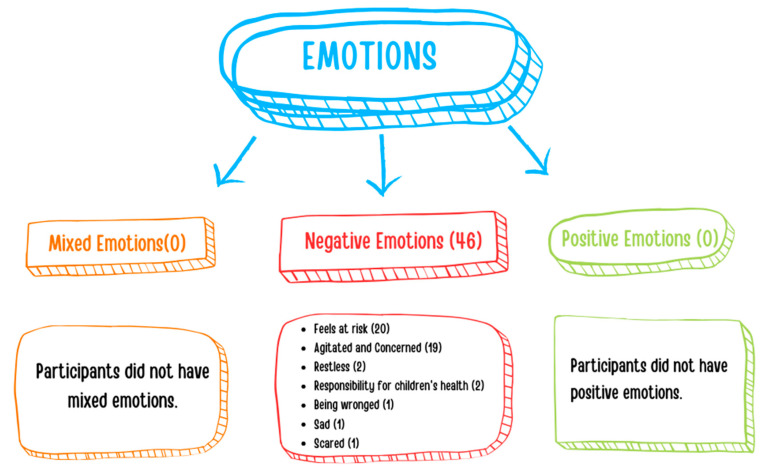
Teachers’ emotions when a child with an infectious disease attends the classroom.

Teachers were asked to describe their emotions when a child with an infectious disease attended the classroom. All of the teachers noted that they experienced negative emotions (n = 46), such as feeling at risk (n = 20), agitated and concerned (n = 19), restless (n = 2), responsible (n = 2), wronged (n = 1), sad (n = 1), and scared (n = 1) (Figure 4).

*“Above all, I feel sorry for the sick child and other students rather than myself. Instead of resting at home and recovering more quickly, the sick student initiates the same cycle for others, thus prolonging the healing process”* (Teacher-18).

**Figure 5:**
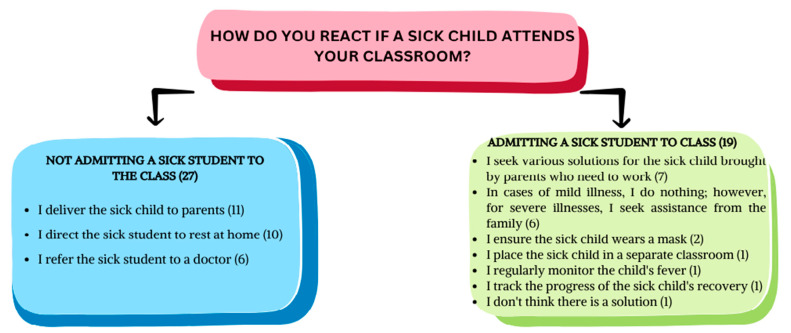
Teachers’ responses when a child with an infectious disease attends the classroom.

Teachers were asked to indicate how they react when a child with an infectious disease attends the classroom. Of the 46 teachers, 27 stated they did not admit a child with an infectious disease into the classroom, while 19 teachers allowed it. Teachers who did not admit children with an infectious disease into the classroom mentioned that they either delivered the sick children to their parents (n = 11), directed them to rest at home (n = 10), or referred them to a doctor (n = 6). It was found that these teachers constantly tried different solutions to prevent transmission and adjusted their behavior according to the severity of the infectious disease (Figure 5).

**Figure 6:**
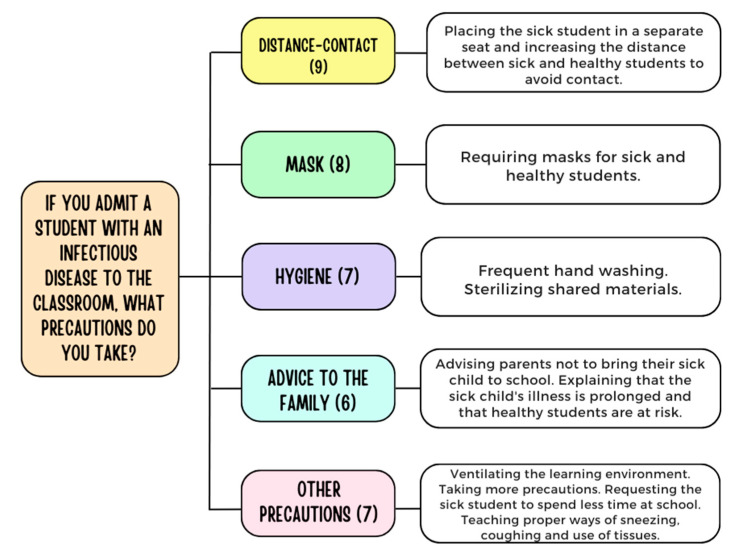
Precautions taken by teachers when a child with an infectious disease attends the classroom.

Teachers (n = 19) who admitted a child with an infectious disease to their classroom were asked to point out what precautions they took. If teachers mentioned multiple precautions, each was included within its own theme. Teachers who allow children with infectious diseases (n = 19) specified 36 measures that were related to the themes of increasing distance-preventing contact (n = 9), requiring masks for sick/healthy children (n = 8), hygiene (n = 7), and advising families (n = 6) and others (n = 7) (Figure 6).

*“In routine meetings, I inform parents about what to pay attention to in institutions, schools, and common areas. I remind them how vital it is for children to rest at home during illness, both for their health and the health of others. If someone has to bring a sick child, I track the progress till recovery”* (Teacher-20).

*“…If parents are not too desperate, they do not bring their children. They consider bringing since they believe their children have recovered. Unless they have fully recovered, I advise the parents to pick up them as soon as possible”* (Teacher-23).

*“I teach the sick child proper ways of coughing and sneezing to prevent the spread of germs”* (Teacher-4).

**Figure 7:**
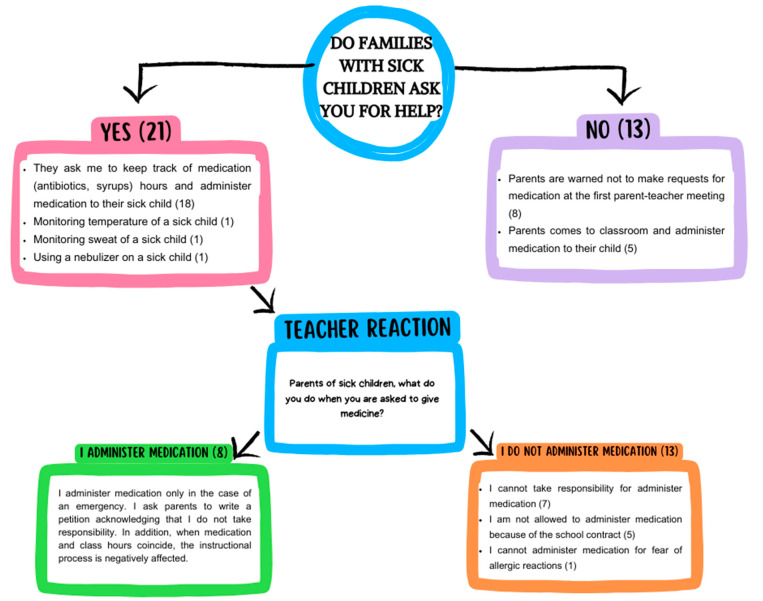
Parents’ requests for assistance from the teacher for their sick children and teacher responses.

Teachers were asked to describe the assistance parents requested for their sick children and how teachers responded to them. Of the teachers who answered this question (n = 34), 21 remarked that parents sought assistance from them, whereas 13 teachers stated that the parents did not ask for help. Parents who sought help from the teachers (n = 21) made requests for their children with infectious diseases regarding medication (n = 18), fever measurement (n = 1), sweat control (n = 1), and nebulizer use (n = 1). Most of them (n = 13) refused to assist and administer medication. In contrast, some (n = 8) indicated that they administered medication to children with infectious diseases. Among the teachers (n = 13) who were not asked for help, eight teachers mentioned that they warned beforehand during the first parent–teacher meeting not to seek assistance for their sick child. Additionally, a few teachers (n = 5) declared that parents came to the classroom and administered medication to their sick children themselves (Figure 7).

### 3.7. Teachers’ Recommendations for the Prevention of Infectious Diseases at Preschool Institutions (Qualitative Data)

**Figure 8:**
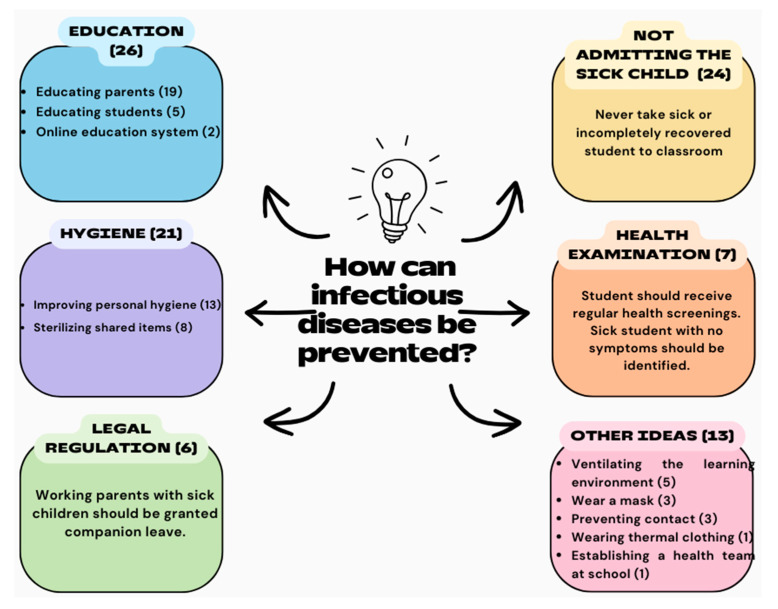
Teachers’ recommendations for the prevention of infectious diseases at preschool institutions.

Teachers were asked to comment on how infectious diseases at preschool institutions can be prevented. If teachers (n = 46) commented on multiple precautions, each was included within its own theme. Teachers stated that infectious diseases could be prevented at preschool institutions through the education of parents and students (n = 26), not admitting a sick child to the classroom (n = 24), hygiene (n = 21), health screening (n = 7), legal regulation (n = 6), and other ideas (n = 13) (Figure 8).

*“My suggestion for a radical solution to the issue is to educate families and children based on the idea that education begins at home. Before their children start school, parents should be given a long-term seminar on topics such as school, school environment, parent-teacher relationship, diseases, and family support for the child, and receive a certificate”* (Teacher-22).

## 4. Discussion

The current study investigated variables concerning teachers, children, parents, and institutions to determine the epidemiology of infectious diseases at preschool institutions, pinpoint the causes of transmission, and explore the possible solutions. In this section, the findings of the research were discussed in accordance with the literature. Alongside that, the authors’ recommendations were integrated within the framework to maintain the integrity of the subject matter.

Most parents participating in the study remarked that their children get sick frequently in the preschool environment. Likewise, studies in the literature identified that the risk of infectious diseases is relatively high at preschool education institutions [5,6,7,8,9,10,11,12,13,14,15,16], which may concern parents. However, their concerns may jeopardize children’s right to access education since, as indicated by some parents in this study, they do not send their healthy children to school if there is a child with an infectious disease at the institution.

Almost half of the parents reported that their children get sick 3–5 times a year, but even though schools have only been open for five months, children have become ill a mean of 3.5 ± 2.0 times. Therefore, children are presumed to get sick more than 3–5 times yearly. It was found that children at private preschool institutions get sick significantly more often than children at public institutions. In Türkiye, public preschool institutions are free, unlike private ones where parents have to pay tuition fees. This fact may be associated with one of the reasons for the higher rate of infectious diseases at private preschool institutions, as parental decisions seem to be affected by the fees paid. Parents paying fees to these institutions presumably expect their children to receive services even when sick in return for their payment. Previous studies have reported that policy factors like fee reimbursement influence parental decisions on sending children with respiratory diseases to daycare [9,16]. This finding corroborates the results of our study. We thus suggest developing policies supporting fee reimbursement for the days children cannot attend school due to sickness. Although public preschool institutions in Türkiye are free, social club activities offered in these schools are subject to fees. Children who are unable to participate in activities for more than seven days within a month due to an illness and similar conditions are refunded the fee for the days when they are absent [29].

Our study uncovered that as the maternal education level increases, children acquire infections more frequently. A previous study found that children whose mothers have higher education levels more frequently experience lower respiratory tract infections compared to children of mothers with lower education levels [6]. As parents’ educational level increases, the employment rate is likely to rise, leading to more involvement in professional life. Consequently, their children spend more time at potentially risky preschool institutions and get sick frequently due to parent-related reasons such as financial and employment pressure. Moreover, parents may transmit infectious diseases from the workplace to the home environment.

More than half of the parents (61.7%) reported that their children had been sick in the last two weeks. In addition, it was discovered that the vast majority of children who got sick (91.5%) used antibiotics and were affected by respiratory tract diseases (91.4%). As stated by Rooshenas et al., antibiotic consumption, usually for viral infections, is the highest among preschool children [13]. Antibiotic-resistant infections are also likely to develop due to unnecessary antibiotic use. This finding, which should be taken seriously with respect to public health, highlights the need for further research on reducing unnecessary antibiotic use. On the other hand, while COVID-19 cases were high globally at the time of the study, the fact that parents did not report COVID-19 cases in children may be related to the rarely seen and mild symptoms of the disease in children [30].

Almost half of the parents in the present study were either not knowledgeable or had limited knowledge about infectious diseases. Parents’ decision-making in sending their kids with infectious diseases to preschool institutions may be flawed due to their lack of understanding about these diseases. Educating parents about them will contribute to protecting and maintaining not only their children’s but also their own health. Therefore, designing intervention programs and training parents about infectious diseases is paramount. The literature has previously highlighted that informing parents who send their children with contagious diseases to childcare centers is crucial to increase awareness about the risks they will face [7].

The current study revealed that the lack of alternative daycare services ranks first among the factors affecting the preferences of parents who send their sick children to school. Alternative daycare services can include establishing isolation classrooms, provided that the institution’s conditions are suitable, or providing alternative classroom options with stringent preventive measures where contact is minimized, thus protecting healthy children from the risk of disease. Another factor influencing parental decision-making is the child’s insistence on attending school despite being ill, which leads to parents taking the sick child to school due to this pressure. Devising alternative options for children with infectious diseases to rest at home and transforming the home environment into a pleasant learning space can alleviate the pressure children may exert on parents. This finding also implied that children should be educated about the potential transmission of certain diseases to their peers. Further, if children persist in desiring to attend school despite their awareness of this risk, it suggests a propensity toward egocentric thought. The literature underscores the necessity of enhancing health education for children at childcare facilities as a means of preventing infections [11].

The majority of parents claimed that they do not send their children to school when they have a contagious disease. Conversely, teachers stated that they frequently attend the classes. What is more, 161 parents reported that they had requested the teacher not to admit children with an infectious disease to the classroom. On the other hand, 119 parents had warned other parents who brought their sick children to school. The findings reported here suggest that children with infectious diseases are frequently present in classrooms. Unless measures are taken, parents whose children are healthy may have disputes with parents sending their sick children to school. In the present study, we discovered that a high proportion of parents do not send their children to school with an infectious disease. Conversely, depending on the responses to other questions and the interviews with teachers, it was determined that too many parents send their children to school regardless of their sickness. There are two possible explanations for why the vast majority of parents participating in this study declared that they do not send their sick children to school. Firstly, this result may be attributed to the possibility that the parents sending their children to school did not want to participate in this study or they constituted a significant portion of those who withdrew from the research. Another possible explanation is that the parents participating in the study did not provide accurate responses due to their hesitation. To overcome this limitation in the research, we conducted interviews with an unbiased approach concerning the presence of children with infectious diseases at schools. Additionally, as an alternative way, we prepared an online interview form and collected data from parents who did not want to participate in a face-to-face interview. Relying on these measures, we believe that our first explanation might be a highly valid possibility that parents who get children with an infectious disease to school may have higher rates of non-participation in this study’s interviews.

This study also identified that when a child with an infectious disease is in the classroom, the majority of parents feel negative emotions, whereas some experience mixed and positive emotions. Likewise, all the teachers indicated that they experienced negative emotions and expressed their feelings as being agitated, concerned, and at risk. In consequence, the presence of a child with an infectious disease in the classroom not only increases the risk of transmission, but also affects the teacher’s mood, adversely affecting the classroom atmosphere and the educational process. This finding suggests that a sick child in the classroom can directly or indirectly influence diverse aspects of an academic environment, such as the teacher’s job satisfaction, the educational process, classroom atmosphere, peer relationships, teacher–child communication, and parent–school relationships. We believe that further studies need to be carried out on this matter.

Another finding to emerge from this study was that as for parents, their children get sick at preschool institutions for a variety of reasons, including contact with another sick person, the presence of a child with an infectious disease in the classroom, inadequate hygiene, insufficient physical classroom environment, bad weather conditions, weak immune system, and unhealthy diet. In response to the question regarding how infectious diseases can be prevented, they provided answers comprehensively covering various themes. Parents believe that preventing infectious diseases at preschool education institutions requires increasing safety measures and precautions, providing education to both parents and children, conducting school health practices, organizing the learning environment, promoting healthy nutrition practices at school, enforcing legal regulations, and ensuring that teachers monitor children who are sweating. Taken together, parents seem to have various expectations from teachers, school management, and policymakers. Teachers, on the other hand, underlined that infectious diseases can be prevented through education, not admitting a sick child to the classroom, hygiene, health screening, legal regulation, and other precautions like masks, ventilation, thermal clothing, preventing contact, and creating a school medical team. It was identified that parents centralize teacher-oriented demands such as enhancing safety measures and precautions for preventing infectious diseases at preschool institutions, regulating the learning environment, monitoring the sweating and fever of sick children, and implementing school health practices. Teachers, by contrast, focus on parent education, exclusion policies for a child with an infectious disease, and improvement in personal hygiene. They also have expectations from policymakers and school management regarding health screenings and legal regulation. The current study has shown that concerning hygiene measures, parents emphasize the need to enhance hygiene in schools, whereas teachers point out the significance of improving personal hygiene practices. According to both parents and teachers, parents sending their child with an infectious disease to school and children should be educated. Therefore, it is vital to educate specifically these parents through official policies and conduct public opinion research. Nonetheless, these trainings should include not only parents but also children, teachers, school administrators, and staff since the lack of education of any component within this system, where the child is at the core, can endanger the health of all individuals involved [31]. On the other hand, in this research, childhood vaccinations, which are the most effective method of protecting against infectious diseases, were not brought to the agenda by either teachers or parents. This may be attributed to the high vaccine acceptance rates in the province where the research was conducted so much so, that in a study conducted in 2021 among parents of children under five years in the Tokat province, the vaccine acceptance rate was found to be 99.1% [32]. In our study, this rate was 97.5%.

In this study, parents and teachers stressed the importance of keeping children with infectious diseases out of the classroom. The presence of a sick child in school for a particular period has recently been expressed in the literature as ‘school-based presenteeism’ [24]. This recent concept highlights the inclination to attend school to avoid absenteeism. Even if we do not consider absence policies as one of the primary reasons preschool children attend school even while sick, they may exert a partial influence due to the fact that students’ attendance status at preschool education institutions in Türkiye is monitored daily and is fundamental [33]. Given that unexcused absences reach 20 days, students are subject to sanctions, yet we can claim that preschool students have flexibility regarding absenteeism. Attendance policies may significantly impact sick students’ attendance at educational institutions, particularly in settings with practical courses, such as universities and high schools. Hence, we assume that school-based presenteeism is primarily directed toward university students, high school students, and those enrolled in practical courses rather than students in preschool education. Consequently, a research gap exists regarding this matter, necessitating further investigation.

Exploring the responses of teachers, pivotal figures in education, on both children with infectious diseases and their parents was imperative for comprehending the results of this investigation. The findings revealed that most teachers do not permit a child with an infectious disease to enter the classroom, while approximately half of the respondents are inclined to admit such children despite their contagious condition. Teachers who did not admit sick children reported that they delivered children to their families and referred them to rest at home or to a doctor. Teachers who permitted a child with an infectious disease to attend classes demonstrated empathy, recognizing the challenges parents face with work commitments. They actively sought a number of strategies to mitigate transmission risks, such as wearing masks, monitoring fever and sweating, monitoring symptoms, and even segregating the sick child into a separate classroom. These are mentioned in the literature as common and effective measures to prevent infectious diseases [34,35]. Moreover, their behaviors were tailored to the severity and nature of the infectious disease. Despite all teachers expressing reluctance to have a child with an infectious disease in their classroom and revealing to experiencing negative emotions when confronted with such a situation, it appears that nearly half of them still allow these children into the classroom, thereby potentially jeopardizing their health as well as that of other students. Accordingly, teachers’ perspectives and behaviors (responses) contradict. Indeed, this discrepancy could be attributed to the teachers’ concerns about maintaining positive relationships and avoiding conflicts with parents, understanding the parents’ troubles, or adhering to directives from the school administration. Teachers who permitted a child with an infectious disease to enter the classroom reported implementing various precautions, including measures to increase physical distance and prevent contact, requiring sick or healthy children to wear masks, enhancing hygiene practices, providing guidance to the family, and implementing additional measures such as improving ventilation, reducing the time the sick child spends in the classroom, and teaching proper coughing etiquette by covering one’s mouth with hands. Such measures reduce the risk of transmission but may have a negative impact on the educational process. These findings suggest that teachers exhibit considerable empathy toward parents who bring their children with infectious diseases to school, despite policies demanding student exclusion and legal regulations. However, their understanding may inadvertently compromise the health of the teachers and other children in the classroom. It is evident that teachers require support as they often feel overwhelmed and isolated and explore effective solutions to this issue independently. There is a pressing need for collaborative efforts to provide them with the necessary support. In addition, it is recommended that further research be undertaken on the quality of relationships and communication between teachers and children with an infectious disease.

In this study, the vast majority of teachers expressed that the parents of sick kids have made some requests to teachers. Teachers who declared experiencing no such requests stated that they warned parents beforehand, so parents visited the classroom during medication hours to administer medicine to their sick children. In that case, the instructional process may be negatively affected if the class and medication hours coincide. Additionally, it was uncovered that children with infectious diseases are occasionally found even in the classrooms of teachers who have implemented rigid rules prohibiting parental requests concerning such cases.

Teachers also noted that parents’ requests regarding their sick children commonly involve administering antibiotics and syrups, measuring fever, monitoring sweating, and using a nebulizer. Most teachers responded negatively to these parental requests, stating their inability to take responsibility for administering medication and other issues due to their concerns about potential allergic reactions and adherence to policies outlined in the school contract. In contrast, some teachers mentioned that they provide medication to sick children but requested parents to draft an agreement or petition acknowledging that the responsibility lies with the family. Moreover, they also commented on the negative impact of the medication hours on the instructional process. On the whole, teacher response is a crucial variable in addressing the prevalence of infectious diseases within preschool institutions, and teachers need assistance and support in this regard.

### Limitations of the Study

The study has several limitations. Since this study evaluated infectious diseases in children at preschool institutions in the Tokat province of Türkiye, the results of the study can only be generalized to this province. One limitation of the study is ‘recall bias’, since some of the information collected through the survey requires participants to recall past events. Finally, a common bias in survey-based studies is social desirability bias, where respondents answer in a way that they assume would make them look better rather than providing honest answers.

## 5. Conclusions

Preschool institutions, of which the significance and enrolment rates are increasing daily, represent risk environments where infectious diseases can spread rapidly. The fact that these institutions are a risk environment is multifaceted, necessitating the consideration of numerous contributing factors. Addressing this risk does not imply completely withdrawing children from preschool education settings. Identifying the relevant risk factors and reasons can contribute to creating a healthier and safer preschool environment. Infectious diseases pose threats beyond individual health and can negatively affect many aspects such as public health, economics, employment, job performance, annual leave duration, antibiotic use, peer relationships, teacher’s mental state, teacher–child relationship, parent–parent relationship, school-parent relationship, and classroom atmosphere. Resolving the rising incidence of infectious diseases at preschool institutions, where children’s personalities are shaped, will foster healthier and better-educated children, who are unquestionably our most valuable assets. Furthermore, it will aid in reducing significantly increased public health risks, healthcare costs, and health disparities.

## Figures and Tables

**Table 1:** Prevalence of infectious diseases in children at preschool institutions and their parents’ views.

Information on Children’s Infectious Diseases and Parents’ Views (n = 397)	n	%
**Frequency of infectious diseases in child within one year**		
1–2 times a year	52	13.1
3–5 times a year	190	47.9
6–8 times a year	107	27.0
9 times a year or more	48	12.1
**Parent’s feeling that their child is sick more often than other children**		
No	208	52.4
Yes	92	23.2
Indecisive	97	24.4
**When does your child tend to get sick more often?**		
Days when the child attends school	229	57.7
It is impossible to distinguish	140	35.3
When the weather turns cold	28	7.1
**The child’s illness after starting preschool education (in almost 5 months)**		
No	16	4.0
Yes	376	94.7
My child was already sick when the school started	5	1.3
**How many times has the child been sick since school started? (n = 376)**		
1–2 times	138	36.7
3–4 times	141	37.5
5–6 times	67	17.8
7–8 times	14	3.7
9–10 times	16	4.3
**Disease frequency (min–max; M ± SD)**	1–10; 3.5 ± 2.0	
**Has the child been sick at all during the last two weeks?**		
No	152	38.3
Yes	245	61.7
**The child’s illness within the last two weeks (n = 245)**		
Respiratory tract infections	224	91.4
Diarrheal diseases	17	6.9
Rash diseases	4	1.6
**Child’s use of medication prescribed by the doctor for this disease (n = 245)**		
No	20	8.2
Yes	225	91.8
**Type of drug used (n = 225)**		
Antibiotics	35	15.5
Antipyretic or painkillers	19	8.5
Antibiotics and antipyretics or painkillers	171	76.0

**Table 2:** Parents’ attitudes and behaviors toward sending their children with an infectious disease to school.

Opinions (n = 397)	n	%
**Do you think you possess sufficient knowledge about childhood infectious diseases?**		
No	31	7.8
Yes	219	55.2
A little	147	37.0
**Do you send your child who has an infectious disease to school?**		
No	378	95.2
Yes	19	4.8
**Reason for sending to school (n = 19)**		
As working parents, there is no one that can provide childcare at home	9	47.4
My child insists on going to school even when he/she is sick (because he/she misses it)	6	31.5
We have to send him to school due to the frequent illnesses experienced by our child	4	21.1
**Requesting the teacher to administer medication to the child (n = 19)**		
No	10	52.6
Yes	9	47.4
**Do you request that the teacher exclude students with ongoing infectious diseases?**		
No	236	59.4
Yes	161	40.6
**Do you warn other parents not to bring students with ongoing infectious diseases to class?**		
No	278	70.0
Yes	119	30.0
**Do you think students with ongoing infectious diseases in the classroom transmit the disease to other students?**		
No	20	5.0
Yes	377	95.0
**Do you think your child is infected at school?**		
No	13	3.3
Yes	323	81.4
Indecisive	61	15.4
**Do you think students with ongoing infectious diseases should attend the class?**		
No	381	96.0
Yes	6	1.5
No idea	10	2.5

**Table 3:** Teachers’ experiences of infectious diseases in children at preschool institutions.

Opinions (n = 46)	n	%
**Do you think you possess sufficient knowledge about childhood infectious diseases?**		
No	6	13.0
Yes	40	87.0
**Do students with ongoing infectious diseases attend the class?**		
No	27	58.7
Yes	19	41.3
**Frequency of students with ongoing infectious diseases attending class**		
Every day	2	10.5
Every week	8	42.1
Every month	9	47.4
**The most common type of infectious disease in students (n = 19)**		
Respiratory infections	16	84.2
Infections with diarrhea	3	15.8
**Do parents bring their children’s disease knowing that they will be infected?**		
All bringing parents know that they will be infected	18	39.1
Some of the parents who bring them in know that they will be infected	26	56.5
None of the parents who bring them in know that they will be infected	2	4.3

## Data Availability

The datasets used and analyzed in the current study are available from the corresponding author upon reasonable request.

## Supplementary Materials

The following supporting information can be downloaded at: https://www.mdpi.com/article/10.3390/children11040447/s1. Table S1: Socio-demographic characteristics of teachers (n = 46); Table S2: Socio-demographic characteristics of parents; Table S3: Some characteristics and health conditions of children; Table S4: Comparison of the frequency of illness of children who got sick after the opening of schools according to some characteristics; Table S5: Comparison of the frequency of sickness of children according to some views of parents of children who got sick after the opening of school on infectious diseases.
