# Supplementary material for: Prevalence of Infectious Diseases in Children at Preschool Education Institutions and Stakeholder Opinions
Source: Children (Basel). 2024 Apr 8;11(4):447. doi: 10.3390/children11040447 (PMC11049231; doi:10.3390/children11040447)
Supplement: Supplementary file 1 [file children-11-00447-s001.zip › children-2946622-supplementary.pdf]

Supplementary Materials

Table S1. Socio-demographic characteristics of teachers (n=46)

**Characteristics** (n=46)	**n**	**%**
**Gender**		
Female	46	100.0
Male	0	0.0
**Age (years)**		
30-34	5	10.9
35-39	13	28.3
40-44	22	47.8
45-49	6	13.1
**Education status**		
Bachelors degree	39	84.8
Master's or doctorate degree	7	15.2
**Working time as a teacher**		
6-10	4	8.7
11 or above	42	91.3
**Classroom type**		
Independent preschool institution	29	63.3
Preschool affiliated with primary school	17	37.0
**Working age group (years)**		
3 age groups	2	4.3
4 age groups	11	23.9
5 age groups	33	71.7
**Age (years) M ± SD**	39.8±4.2	

Table S2. Socio-demographic characteristics of parents.

**Characteristics** (n=397)	**n**	**%**
**Parents**		
Mother	369	92.2
Father	28	7.1
**Mother's age (years)**		
20-29	89	22.4
30-39	261	65.7
40-49	47	11.8
**Father's age (years)**		
20-29	9	2.3
30-39	275	69.3
40-49	106	26.7
50-59	7	1.8
**Education status of mother**		
Primary education	75	18.9
High school	125	31.5
Bachelor's degree	169	42.6
Master's or doctorate degree	28	7.1
**Education status of father**		
Primary education	62	15.6
High school	134	33.8
Bachelor's degree	160	40.3
Master's or doctorate degree	41	10.3
**Mother's profession**		
Housewife	243	61.2
Officer	25	6.3
Teacher-educator/academic	36	9.1
Law enforcement	4	1.0
Worker	36	9.1
Health personnel	38	9.6
Small business	15	3.8
**Father's profession**		
Retired	8	2.0
Officer	56	14.1
Teacher-educator/academic	43	10.8
Law enforcement	36	9.1
Worker	142	35.8
Health personnel	25	6.3
Small business	52	13.1
Freelance	35	8.8
**Economic status**		
Good	29	7.3
Moderate	350	88.2
Poor	18	4.5
**Number of children**		
**1**	119	30.0
2	180	45.3
3	86	21.7
4	10	2.5
5	2	0.5
**Age of mothers (years) M ± SD**	33.4±4.8	
**Age of fathers (years) M ± SD**	37.0±4.8	
**Number of children (number) M ± SD**	1.9±0.8	

Table S3. Some characteristics and health conditions of children.

**Characteristics** (n=397)	**n**	**%**
**Age (years)**		
3	19	4.8
4	72	18.1
5	306	77.1
**Birth order**		
1	187	47.1
2	140	35.3
3	59	14.9
4	11	2.8
**Mode of delivery**		
Normal birth	149	37.5
Cesarean section	248	62.5
**Birth term**		
Maturity birth	310	78.1
Preterm birth	64	16.1
Postmaturity birth	23	5.8
**Breastfeeding time**		
0-6 months	80	20.2
7-12 months	34	8.6
13-18 months	69	17.4
19-24 months	139	35.0
25-30 months	49	12.3
31-36 months	15	3.8
37 months and over	11	2.8
**Chronic disease in child**		
No	368	92.7
Yes	29	7.3
**Distribution of chronic diseases (n=29)**		
Asthma	21	72.4
Epilepsy	4	13.8
Familial Mediterranean Fever	3	10.4
Cerebral palsy	1	3.4
**Allergy in child**		
No	345	86.9
Yes	52	13.1
**Regular and complete status of vaccinations**		
No	10	2.5
Yes	387	97.5
**Mother's smoking while pregnant**		
Never	361	90.9
Rarely	23	5.8
Sometimes	7	1.8
Mostly	6	1.5
**Is there a parent who smokes in the house?**		
No	192	48.4
Yes	205	51.6
**Type of pre-school education institution registered**		
Public school/Free	296	74.6
Private school/Paid	61	15.4
Public school/Paid	40	10.1
**Classroom type**		
Independent preschool institution	199	50.1
Preschool affiliated with primary school	198	49.9
**Age of children (years) M ± SD**	4.7±0.5	

Table S4. Comparison of the frequency of illness of children who got sick after the opening of schools according to some characteristics.

Characteristics (n=376)	n	Mean	SD	t*/F**	p
**Age (years)**					
3	18	3.72	2.052		
4	70	3.81	2.066	0.713	0.491
5	288	3.50	2.080		
**Mode of delivery**					
Normal birth	140	3.51	2.103		
Cesarean section	236	3.60	2.062	-0.375	0.708
**Birth term**					
Maturity birth[a]	294	3.40	1.969		
Preterm birth[b]	62	4.11	2.277	4.634	**0.010**
Postmaturity birth[a.b]	20	4.35	2.540		
**Breastfeeding time**					
0-6 months	77	3.83	2.148		
7-12 months	31	3.81	2.151		
13-18 months	64	3.33	1.968		
19-24 months	131	3.56	2.012	0.725	0.630
25-30 months	47	3.45	2.030		
31-36 months	15	2.93	1.668		
37 months and over	11	3.91	3.270		
**Chronic disease in child**					
No	349	3.52	2.064		
Yes	27	4.19	2.149	-1.612	0.108
**Allergy in child**					
No	326	3.50	2.051	-1.516	0.130
Yes	50	3.98	2.199		
**Regular and complete status of vaccinations**					
No	9	2.78	1.856	0.884	0.249
Yes	367	3.59	2.078		
**Is there a parent who smokes in the house?**					
No	180	3.59	2.134	0.657	0.803
Yes	196	3.54	2.024		
**Type of pre-school education institution registered**					
Public school/Free[a]	279	3.42	2.032		
Private school/Paid[b]	59	4.20	2.164	3.513	**0.031**
Public school/Paid[a.b]	38	3.63	2.111		
**Classroom type**					
Independent kindergarten	194	3.80	2.107	2.255	**0.025**
Preschool affiliated with primary school	182	3.32	2.016		
**Education status of mother**					
Primary education [a]	69	3.28	1.932		
High school [a.b]	118	3.90	2.178		
Bachelor's degree[a]	162	3.31	1.931	3.857	**0.010**
Master's or doctorate degree[b]	27	4.41	2.454		
**Education status of father**					
Primary education	58	3.69	2.062		
High school	124	3.48	2.014		
Bachelor's degree	155	3.52	2.109	0.350	0.789
Master's or doctorate degree	39	3.82	2.199		
**Economic status**					
Good	28	3.71	2.192		
Moderate	331	3.57	2.093	0.372	0.689
Poor	17	3.18	1.510		

**Independent samples t test*

***One-way ANOVA.*

NOTE= Superscripts a and b indicate the difference between groups. There is no difference in groups with the same letters.

Table S5. Comparison of the frequency of sickness of children according to some views of parents of children who got sick after the opening of school on infectious diseases.

Characteristics (n=376)	n	Mean	SD	t*/F**	p
**Thinking you have knowledge about childhood infectious diseases**					
No	30	3.27	1.507		
Yes	207	3.53	2.106	0.599	0.550
A little	139	3.69	2.136		
**Do you ask the teacher to exclude students with ongoing infectious diseases?**					
No	224	3.54	2.051		
Yes	152	3.61	2.116	-0.349	0.727
**Do you warn other parents not to bring students with ongoing infectious diseases to class?**					
No	268	3.54	2.108	-0.429	0.668
Yes	108	3.64	1.997		
**Do you think that students with ongoing infectious diseases in the classroom transmit the disease to other students?**					
No	18	3.17	1.790	-0.838	0.403
Yes	358	3.59	2.088		
**Do you think your child is infected at school?**					
No	12	2.67	1.303		
Yes	307	3.68	2.124	2.698	0.069
Indecisive	57	3.16	1.850		

Independent samples t test

***One-way ANOVA.*

NOTE= Superscripts a and b indicate the difference between groups. There is no difference in groups with the same letters.
